# Latent Tuberculosis in India: An Overview

**DOI:** 10.7759/cureus.35706

**Published:** 2023-03-02

**Authors:** Deepak Vishwakarma, Sudha R Bhoi, Asmita Rannaware

**Affiliations:** 1 Epidemiology and Public Health, Jawaharlal Nehru Medical College, Datta Meghe Institute of Medical Sciences, Wardha, IND

**Keywords:** tuberculosis, immune suppression, latent tuberculosis, dormancy, persistence, including latency

## Abstract

Treatment of latent tuberculosis infection (LTBI) is essential for tuberculosis (TB) eradication. LTBI patients serve as a reservoir for active TB cases. The WHO's End TB Strategy now prioritises the detection and treatment of LTBI. A comprehensive approach focused on integrated LTBI control is required to accomplish this goal. This review aims to summarise what we know about LTBI in the existing literature, its prominence, diagnostic strategies, and new interventions to alert people of its occurrence and symptoms. We used Medical Subject Heading (MeSH) phrases to search for published publications on the English language in PubMed, Scopus, and Google Scholar. To provide clarity and impact, we examined several government websites to identify the most effective and current treatment regimens. LTBI is a spectrum of infections, such as intermittent, transitory, or progressive, with early, subclinical, and ultimately active TB cases. The global burden of LTBI cannot be firmly established because no “gold-standard” test exists. Screening is advised for high-risk individuals, such as immigrants, occupants and staff members of congregate living facilities, and those who are HIV-positive. The most reliable form of LTBI screening is still the targeted tuberculin skin test (TST). Although LTBI therapy is challenging, for India to become TB-free, it must first focus on testing and treating LTBI. The government should focus on generalising the new diagnostic criteria and adopting a more specific treatment known to all to eliminate TB once and for all.

## Introduction and background

According to estimates, almost 33% of people worldwide have latent tuberculosis infections (LTBI). Although estimates for LTBI prevalence in India's population are unavailable, according to the WHO estimates, approximately 3.5 lakh children under five are qualified for LTBI treatment [1]. Tuberculosis (TB) kills 1.5 million people annually, having a mortality rate of more than any other infectious disease. The chance of having TB disease increases after infection, and clinical manifestations peak in the first two years after acquiring infection, which can last a lifetime. According to typical estimates, “one-third” of the world's population, or roughly 2.3 billion people, have LTBI [2-4]. The WHO estimates that approximately 2 billion people, or more than one-third of the global total, are TB carriers [5]. People with latent TB do not exhibit symptoms. A person with latent TB can be a carrier of TB by activating the disease agent in the body. Thus, people with LTBI should receive treatment to stop them from acquiring TB illness [3].

Due to the lack of symptoms associated with LTBI, patients cannot spread the disease until the Mycobacterium becomes active [6]. Most individuals with high immunity affected by LTBI due to airborne infection can typically resist those bacteria and prevent them from multiplying. Patients with LTBI usually respond positively to a skin test for TB. A person with LTBI will test positive for a TB blood test and not exhibit symptoms [6]. LTBI is the continuance of immune responses, following antigen stimulation of Mycobacterium tuberculosis (Mtb) that occurs without clinically active disease.

If left untreated, 510% of patients having LTBI will gradually develop TB disease throughout their lives [3]. The transition phase from LTBI to active TB infection can linger long. TB disease can be fatal if not aptly treated [6]. The main focus of public health initiatives to combat TB has been detecting active cases. However, to achieve these challenging goals, modelling suggests that it is essential to decrease the LTBI reservoir with preventive medication. To significantly reduce TB transmission, a halt for LTBI development into active TB is necessary. Targeted treatment of infected persons at risk of developing active TB disease is a crucial component of the End TB Strategy [7].

Currently, the WHO's prioritised areas of the End TB Strategy are the detection and management of LTBI. This review aims to summarise what we know about LTBI in the existing literature, its prominence, diagnostic strategies, and new interventions to alert people of its occurrence and symptoms. The reason is that those who have LTBI run a considerably higher risk of developing active TB or having it reactivated than healthy individuals. Following international strategies like the WHO's End TB Strategy targets, Sustainable Development Goals, and the national strategic plan present audacious measures involving proportionate resources to eradicate TB in India by 2030 [8]. This article will be valuable for public health professionals and policymakers who work with infectious diseases, such as TB, and students who wish to learn more about LTBI.

## Review

Methodology

**Figure 1:**
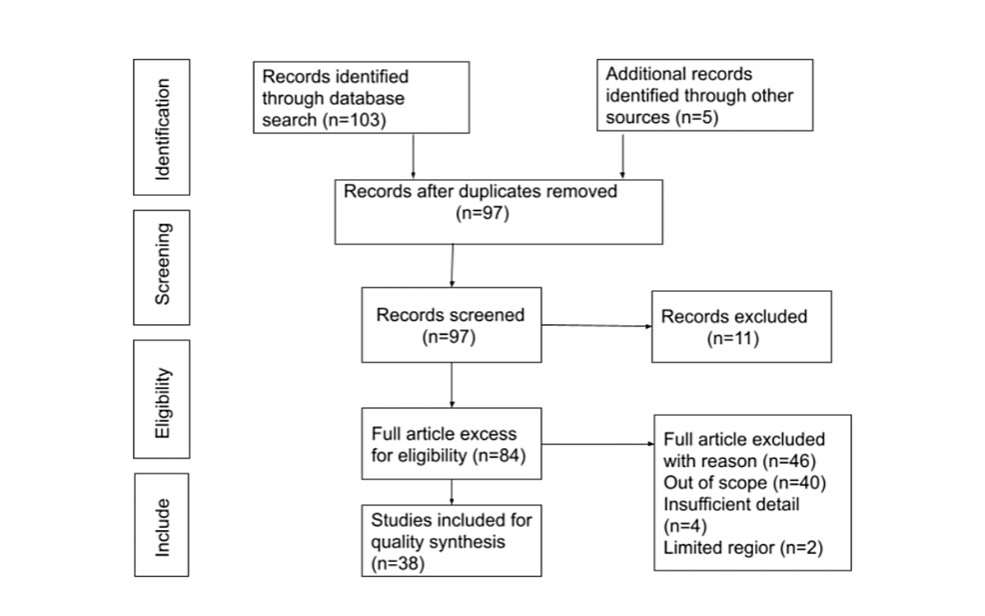
Inclusion and exclusion criteria Self-made

We used Medical Subject Heading (MeSH) phrases to search for published publications in the English language on PubMed, Scopus, and Google Scholar, including ''latent tuberculosis,” “tuberculosis,” “dormancy,” "persistence," “latency,” “immune suppression,” and Boolean operators like “AND/OR.” We included a range from 2002 to 2022 studies on LTBI in India and its treatment and diagnostic criteria. The inclusion criteria followed articles focused solely on LTBI, its pathology, and the treatment required. Figure 1 depicts the criteria for inclusion and exclusion.

Discussion

According to estimates, 33% of the world's population may have Mycobacterium tuberculosis infection. However, the global burden of LTBI cannot be firmly established because no “gold-standard” test exists. LTBI, a clinical condition, is identified in patients who tested positive using TSTs but with no radiological or clinical symptoms of active disease [9]. HIV/AIDS TB infection accounts for about 25% of HIV-related mortality. According to several studies, LTBI activation risk may increase 10 to 110 times in those who have HIV [10]. Numerous fundamental difficulties concerning the mechanics of latency remain unanswered, although Mtb can exist in a latent or dormant state in human tissue [11]. If all of these people with LTBI are discovered and receive complete treatment, this can lower the number of current TB cases in a community by up to 80-90% [12].

However, the situation is different in high-TB prevalence regions, such as India. The prime goals of all TB control initiatives continue to be the effective diagnosis and treatment of people with active TB, neglecting the ones who might have LTBI. Active TB treatment is the top goal of any TB control program. Including LTBI in the national program will make the health system more logistically and financially burdened [13]. Some studies show that a short course of Isoniazid (INH) was shown to help treat disease and prove effective in preventing illness in guinea pigs and humans [14]. Effective treatment for LTBI prevents the disease from developing into active TB [15]. Despite its efficient antimicrobial therapy, TB is still the most common infectious disease, which results in death, which is of global concern. Various preventive measures must be applied to prevent this mortality of concern [16].

Traditionally, LTBI occurs when the bacilli in previous lesions stay in a non-replicating condition (dormant). However, they can still trigger reactivation, resulting in active TB once the immune response is disrupted [17]. Several variables raise the chance of getting active TB in people with LTBI. Most of them are associated with a weakened immune system, including diabetes, cancer, immunosuppressive medication, and co-morbidity with HIV [18]. HIV and TB can combine to shorten lifespan if untreated, just like other opportunistic illnesses. An individual with untreated LTBI and HIV infection has a much higher lifetime probability of getting TB than someone who does not have HIV [6].

Natural History of Tuberculosis

**Figure 2:**
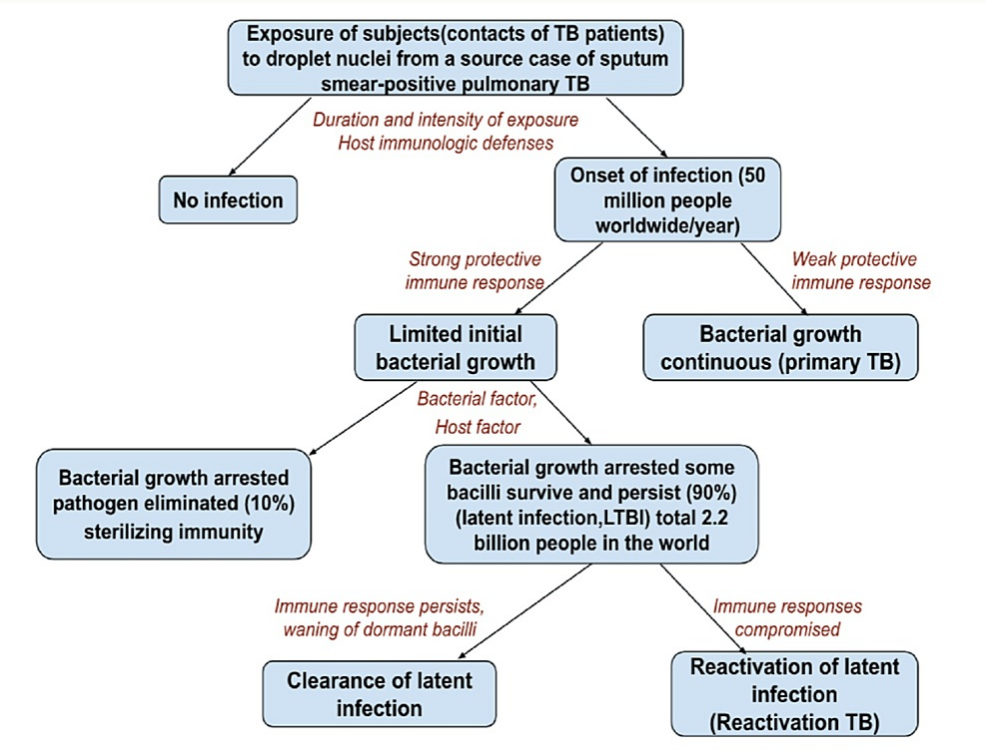
Natural history of TB and progression of TB to LTBI Data Source [19]. Source: Open access journal under a CC-BY license contributed by Suhail Ahmad, Department of Microbiology, Faculty of Medicine, Kuwait University, Jabria, Kuwait. Ahmad S: New approaches in the diagnosis and treatment of latent tuberculosis infection. Respir Res. 2010, 11:169. 10.1186/1465-9921-11-169 TB, tuberculosis; LTBI, latent tuberculosis infection

Figure 2 demonstrates the progression of symptoms and prognosis in a healthy person after exposure to Mtb droplet nuclei which are spread spontaneously from a source case of sputum smear-positive lung TB in study participants (contacts of TB patients) [19]. Around 50 million people around the world contract Mtb each year. Only 10% of patients completely eradicate tubercle bacilli, while 90% of patients with infection cease bacterial growth. Mtb bacilli that survive persist as LTBI. In LTBI patients, treatment using isoniazid (INH) over nine months could hasten the decrease of latent bacilli. The vaccines for LTBI are now undergoing clinical trials, primarily intended to stop or postpone the latent infection in individuals who already have LTBI [19].

Screening for LTBI

Screening is essential for initiating treatment. The decision for treatment is made after TB screening. Individuals at risk of getting Mtb and progression from LTBI to active TB benefit from screening for LTBI. Routine testing outside these high-risk areas is resource-wasting and raises the risk of false-positive test results. The targeted Mantoux tuberculin skin test (TST) is the most extensively used tool for LTBI screening [20]. Improved TB diagnostics are necessary to stop and reverse the growing global disease burden [21].

Recommendations for Testing of LTBI

A sensitive and precise LTBI diagnosis is required to manage prophylactic treatments efficiently. The gold standard for diagnosing active infection is bacterial culture, but there is no equivalent standard for detecting LTBI [7,22]. Diagnosis of LTBI includes either an interferon-gamma release assay (IGRA) or a TST [23]. In higher-middle-income and high-income countries, the WHO recommendation for LTBI guidelines is either IGRA or TST for LTBI testing; however, IGRA should not take the role of TST in lower-middle and low-income countries. According to the WHO post-2015 End TB Strategies recommendation for the management of LTBI for higher/upper middle-income countries with TB incidence of less than 100/100,000 individuals, systematic testing and treatment for LTBI is strongly recommended [24].

**Figure 3:**
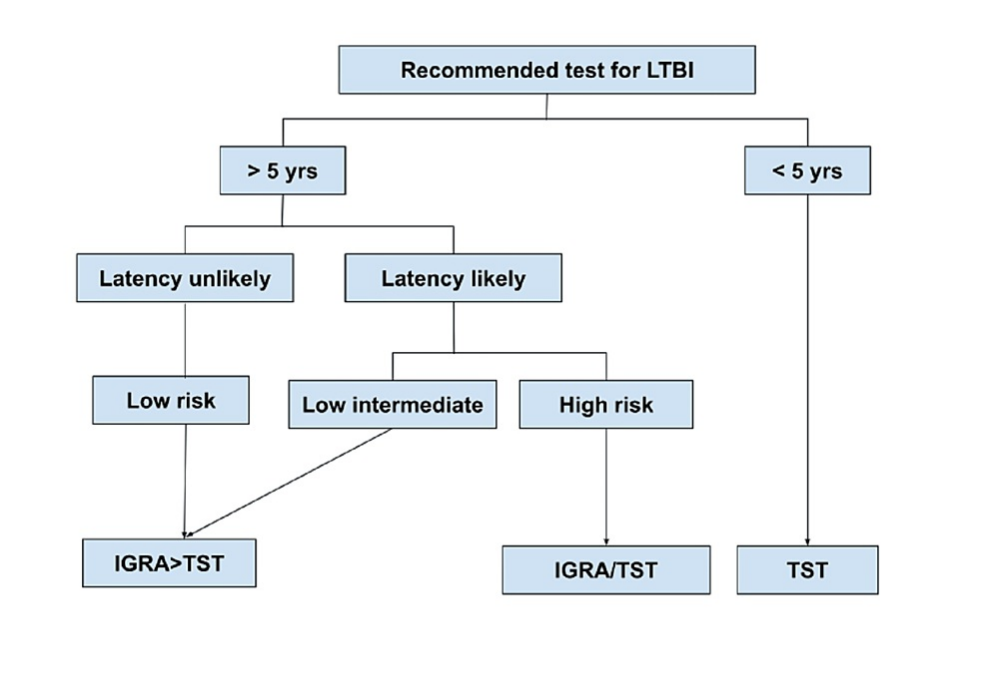
Method for testing for LTBI (modified from WHO and CDC guidelines). In India, the TST may be chosen over the IGRA Adapted from [1]. Source: Open access journal under a CC-BY license Contributed by Dr. A. Kumar, Department of Medicine, All India Institute of Medical Sciences, Teaching Block, 3094A, Ansari Nagar, New Delhi. Saha S, Kumar A, Saurabh K, et al.: Current status of treatment of latent tuberculosis infection in India. Indian J Med Sci. 2020, 71:54-9. 10.25259/IJMS_18_2019 LTBI, latent tuberculosis infection; TST, tuberculin skin test; IGRA, interferon-gamma release assay

Figure 3 depicts a flowchart format for recommended tests for LTBI [1].

New LTBI Diagnostics

Due to the deficit of the accepted gold standard for diagnosing LTBI, there may be problems with outcome classification [25]. The development of new LTBI diagnostics is gradual. The C-Tb testing is one of the most recent advancements, which employs M antigens that are unique to TB, like those used in IGRAs, which maintain the sensitivity of the current TST assay while removing cross-reactivity with the Bacille Calmette-Guérin vaccination [26]. The world has just launched a new version of the QuantiFERON test. New antigens have been incorporated into the QuantiFERON-TB Gold Plus assay to improve test sensitivity in immunocompromised populations, including HIV-positive individuals. However, the early independent evaluations revealed that the sensitivity of this new test has increased slightly [27]. Aside from sensitivity issues, neither the IGRA nor the TST can reliably differentiate between active TB disease and LTBI, nor can they predict LTBI reactivation. The field of LTBI diagnostics needs to advance to create better instruments for diagnosing LTBI and forecasting LTBI reactivation [7].

Mechanism

According to conventional wisdom, latent bacilli remain dormant within early lesions within the upper lobes of the lung. Individuals with LTBI kept them for the rest of their lives. As a result of the reactivation of these dormant bacilli, TB develops because of resuscitation factors. Furthermore, the pressure within the upper lobes increases, promoting bacillary multiplication and lessening immunological responses [28].

**Figure 4:**
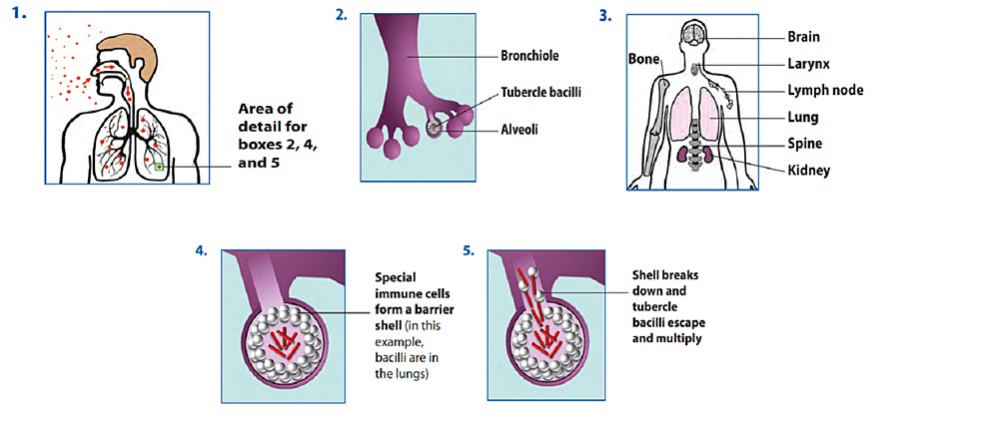
Pathogenesis of TB and LTBI disease Adapted from [29]. Source: Open access journal under a CC-BY license Contributed by CDC. Centers for Disease Control and Prevention: Module 1: Transmission and Pathogenesis of Tuberculosis. CDC (ed): CDC, Atlanta, Georgia; 2019.

The disease develops when a person breathes tubercle bacilli-containing droplet nuclei that reach the lungs' alveoli. Alveolar macrophages consume these tubercle bacilli; most bacilli are killed or inhibited. A small percentage of the dead macrophages may multiply intracellularly and eject themselves. If these bacteria are alive and well, they might spread to other tissues and organs via the lymphatic or circulatory systems (including areas where TB disease is most likely to develop apex of the lung, kidneys, bone, regional lymph nodes, and brain). The immune system is prepared for a widespread reaction by this dissemination process. The pathogenesis of LTBI is demonstrated below in Figure 4 [29].

Treatment

The long-term reduction of the TB burden can be achieved in two ways. One strategy calls for the prompt and early detection of all individuals having active TB and the implementation of appropriate treatment to render them non-contagious. As a result, fewer carriers will later acquire active TB, limiting transmission [5]. An alternative strategy is to identify those still suffering from LTBI and treat them to stop the later onset of the disease. The government first used this treatment strategy in the 1950s. Clinicians noticed that INH monotherapy effectively suppressed disease progression in children with primary TB soon after Isoniazid (INH) was introduced as a treatment for the disease [5,30]. The trials showed that people with LTBI who took INH for at least six months saw a 25-92% reduction in the incidence of TB in the future [5].

However, there are several issues with INH treatment for LTBI. The prolonged course of therapy decreases patient compliance, and the possibility of significant adverse effects, including hepatitis, further discourages patients and healthcare professionals from accepting this therapy. Therefore, shorter alternative regimens have received a lot of attention and analysis [5]. Evidence suggests that six months of INH prophylactic LTBI treatment increases the risk of TB reactivation and reinfection [31]. As a result of the preceding, before beginning LTBI therapy, those at risk of developing TB should undergo a clinical examination.

Additionally, it is evident that to eradicate TB, it will be necessary to develop new, safer medications for LTBI treatment that providers can give for shorter periods and suitable biomarkers to gauge the treatment's effectiveness [18]. Using an animal model of mycobacterial infection, a study conducted in this context found that INH therapy reduces the number of microorganisms in the body. Still, INH-treated animals show significant suppression of the antigen-specific proliferating response compared to untreated mice [32].

Treatment providers should check for active TB before starting LTBI treatment using the history, physical examination, chest radiography, and, if necessary, bacteriologic investigations [32]. LTBI treatment needs lengthy chemotherapy (9 months), which makes treatment compliance very challenging [33]. Isoniazid is sometimes ineffective against persisters but still the most often prescribed medication for chemoprophylaxis. Studies have established that treating LTBI prevents active disease, regardless of the mechanism through which TB becomes latent [15].

In latent TB infections, the bacillary load is low; therefore, tubercle bacilli are typically non-recoverable from sputum and other places of a TB disease individual. LTBI treatment involves nine months of Isoniazid (INH), which blocks the pathway of production of mycolic acid (needed for cell walls), and Rifampin (a transcriptional inhibitor) for four months, or two months of Rifampin and Pyrazinamide, a sterilising drug that targets dormant bacilli living in an acidic environment [34-37]. Table 1 demonstrates the drug dosage recommendations for LTBI treatment by the Centers for Disease Control and Prevention (CDC) [38].

**Table 1:** Drug dosage recommendations for LTBI treatment Data Source [38].

Regimen	Duration	Adults’ dose/body weight (kg)	Children’s dose/body weight (kg)	Maximum dose
Isoniazid monotherapy	6 or 9 months, daily	5 mg	10 mg (range 7-15 mg)	300 mg
Rifampicin monotherapy	3–4 months, daily	10 mg	15 mg (10-20 mg)	600 mg
Isoniazid +Rifampicin	3–4 months, daily	5 mg isoniazid	10 mg isoniazid (range 7-15 mg)	Isoniazid 300 mg
		10 mg Rifampicin	Rifampicin 15 mg (range, 10-20 mg)	Rifampicin 600 mg
Rifapentine +Isoniazid	Weekly for three months (12 doses)	Isoniazid		Isoniazid 900 mg
		Aged ≥12 years=15 mg		
		2-11 years=25 mg		
		Rifapentine		Rifapentin 900 mg
		10-14 kg=300 mg		
		14.1-25 kg=450 mg		
		25.1–32 kg=600 mg		
		32.1-50 kg=750 mg		
		>50 kg= 900 mg		

## Conclusions

TB continues to be a significant public health issue in every country worldwide. Therefore, all the national programmes are presently concentrated on treating active TB disease. However, this strategy alone cannot eliminate TB. Tracking LTBI cases can be an effective intervention for TB elimination. Shorter and even more efficient preventive and therapeutic interventions, innovative tests, and higher sensitive LTBI diagnostics will be needed to help diagnose most at-risk patients for TB reactivation. Five per cent to 10% of LTBI patients who are not treated will acquire active TB throughout their lifetime. Although LTBI therapy is challenging, if India is to become TB-free, we must first focus on testing and treating LTBI. Additionally, it is clear that to eradicate TB, it will be necessary to create new, safer drugs for LTBI treatment that healthcare professionals may administer for shorter periods and appropriate biomarkers to determine the treatment's efficacy.

The treatment of active disease is the main objective in developing nations with high TB prevalence, like India. A significant part of the TB elimination campaign should include a focus on treating LTBI to decrease the likelihood of active TB. The government should focus on generalising the new diagnostic criteria and adopting a more specific treatment known to all to eliminate TB once and for all.
